# Acute Glucagon Induces Postprandial Peripheral Insulin Resistance

**DOI:** 10.1371/journal.pone.0127221

**Published:** 2015-05-11

**Authors:** Rita S. Patarrão, W. Wayne Lautt, M. Paula Macedo

**Affiliations:** 1 Centro de Estudos de Doenças Crónicas, CEDOC, NOVA Medical School / Faculdade de Ciências Médicas, Universidade Nova de Lisboa, Campo dos Mártires da Pátria, Lisboa, Portugal; 2 Department of Pharmacology and Therapeutics, Faculty of Medicine, University of Manitoba, Winnipeg, Manitoba, Canada; 3 Centro de Estudos de Doenças Crónicas, CEDOC, NOVA Medical School / Faculdade de Ciências Médicas, Universidade Nova de Lisboa, Campo dos Mártires da Pátria, Lisboa, Portugal; 4 APDP-ERC Portuguese Diabetes AssociationEducationand Research Center, Rua do Salitre, Lisboa, Portugal; State University of Rio de Janeiro, Biomedical Center, Institute of Biology, BRAZIL

## Abstract

Glucagon levels are often moderately elevated in diabetes. It is known that glucagon leads to a decrease in hepatic glutathione (GSH) synthesis that in turn is associated with decreased postprandial insulin sensitivity. Given that cAMP pathway controls GSH levels we tested whether insulin sensitivity decreases after intraportal (ipv) administration of a cAMP analog (DBcAMP), and investigated whether glucagon promotes insulin resistance through decreasing hepatic GSH levels.Insulin sensitivity was determined in fed male Sprague-Dawley rats using a modified euglycemic hyperinsulinemic clamp in the postprandial state upon ipv administration of DBcAMP as well as glucagon infusion. Glucagon effects on insulin sensitivity was assessed in the presence or absence of postprandial insulin sensitivity inhibition by administration of L-NMMA. Hepatic GSH and NO content and plasma levels of NO were measured after acute ipv glucagon infusion. Insulin sensitivity was assessed in the fed state and after ipv glucagon infusion in the presence of GSH-E. We founf that DBcAMP and glucagon produce a decrease of insulin sensitivity, in a dose-dependent manner. Glucagon-induced decrease of postprandial insulin sensitivity correlated with decreased hepatic GSH content and was restored by administration of GSH-E. Furthermore, inhibition of postprandial decrease of insulin sensitivity L-NMMA was not overcome by glucagon, but glucagon did not affect hepatic and plasma levels of NO. These results show that glucagon decreases postprandial insulin sensitivity through reducing hepatic GSH levels, an effect that is mimicked by increasing cAMP hepatic levels and requires physiological NO levels. These observations support the hypothesis that glucagon acts via adenylate cyclase to decrease hepatic GSH levels and induce insulin resistance. We suggest that the glucagon-cAMP-GSH axis is a potential therapeutic target to address insulin resistance in pathological conditions.

## Introduction

The prandial status modulates the physiology of whole-body insulin-stimulated glucose disposal which reaches a maximum after a meal and decreases by about 55% after a 24h fasting period [1, 2]. Maximal postprandial insulin sensitivity requires signals provided by the hepatic parasympathetic nerves (HPN) [1] and it was recently demonstrated that HPN controls postprandial plasma glucose clearance by skeletal muscle, heart, and kidney [3]. HPN dysfunction accounts for a 40% reduction of plasma glucose clearance, mainly due to a reduced uptake by skeletal muscle [3]. HPN increase hepatic nitric oxide (NO) [4]. The HPN-NO signal maximizes postprandial insulin sensitivity in presence of optimal levels of hepatic glutathione (GSH) [5]. We have shown that hepatic GSH levels increase in the postprandial state and that co-administration of NO and GSH during fasting induces maximal postprandial insulin sensitivity [5]. Together these data strongly suggest that GSH and HPN-NO interplay in the liver to achieve maximal peripheral postprandial insulin action. On the other hand, it is well known that cAMP is a negative regulator of hepatic GSH production [6–11] but it remains unknown whether this action impacts on postprandial insulin sensitivity.

Glucagon is a pancreatic hormone released in the fasted state in order to maintain an adequate blood glucose level. According to Lu and colleagues glucagon also regulates cAMP that acts in the liver to decrease hepatic GSH levels [6]. This glucagon effect is achieved through increasing hepatic cAMP levels that inhibits γ-glutamylcysteine synthase, a key player in the GSH synthesis [6, 11]. Thus, during fasting, when glucagon levels are high, GSH levels are low but after feeding, glucagon levels decrease and hepatic GSH increases. These observations are consistent with human physiology where plasma glucagon levels increase with fasting and decrease in the immediate postprandial state [12, 13] while GSH shows the inverse pattern [14, 15]. However, in patients with type 2 diabetes, plasma levels of glucagon remain abnormally high after ingestion of a meal and may contribute to impaired glucose tolerance further suggesting that the cAMP signaling pathway is upregulated in insulin-resistant individuals [12, 16, 17]. In fact, hyperglucagonemia has also been associated with hyperglycemia and hyperinsulinemia but the mechanism underlying hyperinsulinemia/hyperglucagonemia inability to counteract the insulin resistance state and its relation with hepatic GSH levels is unrevealed [18].

Here, we tested the hypothesis that glucagon modulates hepatic GSH content, through the activation of the adenylate cyclase pathway, resulting in a state of postprandial insulin resistance.

## Methods

### Presurgical Protocols

Male Sprague-Dawley rats weighing 319.4±7.6g (9-weeks old) from Charles River, St. Constant, Quebec, Canada were maintained in the animal house under controlled conditions (22±1°C) on a 12h light/dark cycle. Rats had *ad libitum* access to standard rat chow diet (Prolab RMH 3000 5P00, Labdiet, USA) and with free access to normal tap water, for one week to adapt to the housing environment.

The animals were kept anesthetised during the experiment and at the end of the protocols they were euthanized with a lethal injection of sodium pentobarbital in accordance with the guidelines of the Canadian Council on Animal Care (CCAC).

Rats were anesthetized with an intraperitoneal injection of sodium pentobarbital (65mg/kg) and anesthesia was maintained throughout the experiment by continuous infusion into the femoral vein (10mg/h/kg). During all the surgical procedures, body temperature was monitored with a rectal probe and kept at 37.0±0.5°C, by means of a heated surgical table (Harvard Apparatus, Kent, England) and overhead lamp.

All the animals were treated according to the guidelines of the CCAC, and the ethics committee on animal care at the University of Manitoba approved all protocols.

### Surgical Protocol

The trachea was cannulated (polyethylene tubing, PE 240, Becton Dickinson, USA) to allow spontaneous respiration. The femoral artery and the femoral vein were cannulated (polyethylene tubing, PE 50, Becton Dickinson, USA) to establish an arterial-venous shunt [19], primed with heparin (200 UI/ml). The jugular vein was cannulated (polyethylene tubing, PE 50, Becton Dickinson, USA) to infuse glucose. After laparotomy, the portal vein was also cannulated(24G Optiva, iv catheter radiopaque ocrilon polyurethane, 19mm, Johnson & Johnson Medical Inc., Arlington, TX). Blood pressure was continuously monitored by a pressure transducer (National Instruments LabView, Austin, USA). A data acquisition system (National Instruments LabView, Austin, USA) combined with application software was used to record and analyze the mean arterial blood pressure. By clamping the venous outlet of the arterial-venous shunt, mean arterial pressure (MAP) was determined.

Rats were allowed to stabilize from the surgical intervention for at least 30minutes before any procedures were carried out. After stabilization, arterial blood samples (25μl) were collected every 5minutes, and glucose concentration was immediately determined, by the glucose oxidase method using a glucose analyser (1500 YSI Sport, Yellow Springs Instruments, USA), until three successive stable glucose measures were obtained. The mean of these three values is referred to as the basal glucose level.

Drugs were administered either intravenously (iv), by puncturing the shunt on the venous side or through the jugular catheter, or intraportally (ipv), through a portal vein catheter.

### Rapid Insulin Sensitivity Test (RIST)

The methodology chosen to evaluate insulin sensitivity was the Rapid Insulin Sensitivity Test (RIST), previously described [19]. The RIST started with the administration of an iv insulin bolus (50mU/kg), over 5minutes, using an infusion pump (Genie, Kent Scientific Corporation, Litchfield, Massachusetts). One minute after starting the insulin administration, a glucose infusion (D-Glucose, 100mg/ml, iv) was started at a rate of 2.5mg/kg/min,to avoid hypoglycemia. Arterial glucose levels were measured at 2-min intervals, and the rate of glucose infusion was adjusted in order to maintain euglycemia. When no further glucose was required, the test was concluded. The amount of glucose infused during the test is referred to as the RIST Index (mg glucose/kg bw) and it is the parameter used to evaluate total insulin sensitivity.

### Hepatic glutathione levels

The medial and the right lobes of the rat liver were harvested before sacrificed the animal, and immediately frozen using dry ice, for glutathione quantification. The liver samples were wrapped and stored in -80°C freezer. The hepatic glutathione was measure using the glutathione assay kit BIOTECH GSH-420, which is a quantitative and colorimetric kit for determination of total glutathione (BIOXYTECH and OxisResearch,OXIS International, Inc. Portland).

### Plasma and hepatic nitric oxide levels

Plasma nitric oxide levels were determined by the chemiluminescence technique, using a Sievers 280 NO Analyzer (Sievers Instruments) as previously described [20, 21]. The medial and the right lobes of the rat liver were harvested before sacrificed the animal, and immediately frozen using dry ice, for nitric oxide quantification. The liver samples were wrapped and stored in -80°C freezer. The hepatic nitric oxide was measure using a Sievers 280 NO Analyzer (Sievers Instruments).

## Experimental Protocols

### 1. Hepatic effect of DBcAMP, a cAMP analogue, on insulin sensitivity

After a control RIST in the fed state, DBcAMP (N^6^,2^’^-O-dibutyryladenosine 3’,5’-cyclic monophosphate), a cAMP analog, was infused ipv at different doses ranging from 0.01 to 1.0mg/kg, for 10minutes at an infusion rate of 0.04ml/min. The second RIST was carried out after DBcAMP ipv infusion.

### 2. Hepatic effect of glucagon, on postprandial insulin sensitivity

This protocol was divided into 2 different series. In the first series, after a control fed RIST, glucagon was infused ipv for 10minutes at an infusion rate of 0.04ml/min, up to a final dose that ranged from 0.5ng/kg to 20μg/kg,. A second RIST was carried out after glucagon infusion.

In the second series, after a control fed RIST, L-NMMA (N-monomethyl-L-arginine) was infused ipv at a dose of 0.73mg/kg for 10minutes at an infusion rate of 0.04ml/min. The second RIST was carried out after L-NMMA ipv infusion. Then, glucagon was infused ipv at 200ng/kg for 10minutes at an infusion rate of 0.04ml/min, and a third RIST was carried out.

### 3. Hepatic glutathione levels in the fasted and fed states, and after ipv glucagon 200ng/kg infusion

In one set of experiments, the livers of fed and 24h fasted animals were used as controls, for hepatic GSH measurement. In other set of experiments, glucagon was infused ipv at 200ng/kg for 10minutes at an infusion rate of 0.04ml/min and 30minutes after glucagon infusion the liver was harvested, for hepatic GSH measurement. The liver samples were wrapped and stored in -80°C freezer until further analysis.

### 4. Plasma and hepatic nitric oxide levels in the fed states and after ipv glucagon 200ng/kg infusion

In one set of experiments, the livers and plasma of fed animals were used as controls for NO measurement. In other set of experiments, glucagon was infused ipv at 200ng/kg for 10minutes at an infusion rate of 0.04ml/min and 30minutes after glucagon infusion the liver was harvested and blood was collected for hepatic and plasma NO measurement, respectively. The liver samples were wrapped and plasma samples were stored in -80°C freezer until further analysis.

### 5. Hepatic effect of glucagon in a presence of a glutathione analog

After a control fed RIST, GSH-E (Glutathione monoethylester) 1mmol/kg was administered ipv as a 10minutes bolus. After a 20min period of stabilization, glucagon was infused ipv at 200ng/kg for 10minutes at an infusion rate of 0.04ml/min. Thirty minutes after glucagon infusion, a second RIST was performed.

### Drugs

Sodium pentobarbital (Somnotol) was obtained from Biomeda-MTC Animal Health Inc., Cambridge, Ontario. Human insulin (NovolingeToronto) was purchased from Novo Nordisk (Mississauga, ON, Canada). Heparin was purchased from Pharmaceutical Partners of Canada, Richmond Hill, Ontario and saline from Baxter Corporation, Toronto, Ontario, Canada. D-Glucose, L-NMMA, DBcAMP and glucagon were purchased from Sigma Chemical Co. (St. Louis, MO, USA). GSH-E was purchased from Bachem, Switzerland. Tissue adhesive was acquired from GluStich Inc., Canada. All chemicals were of the highest degree of purity on the market. All the solutions for *in vivo* administration were prepared in NaCl 0.9%.

### Data analysis

The statistical analysis was performed by paired t-student test, two-tailed. One way ANOVA, repeated measures ANOVA, followed by Tukey's Multiple Comparison Test. Differences were accepted as statistically significant at *p*<0.05. Whenever *p* value is not indicated, differences are not statistically significant. Data were expressed as mean±SEM.

## Results

### Hepatic cAMP effects on postprandial insulin sensitivity

Hepatic cAMP regulates GSH levels that in turn control insulin sensitivity. We tested whether intraportal administration of a cAMP analogue (DBcAMP) impacts on postprandial insulin sensitivity in rats.

Firstly, we check that the DBcAMP doses used did not change mean arterial pressure (Table 1). Moreover, in the fed state, DBcAMP 0.01mg/kg infusion did not affect glycemic levels, while 0.1 and 1mg/kg doses induced transitory increased glycemia levels (Table 1, S2 Fig), that were resolved at the time of insulin sensitivity measurements (post-DBcAMP RIST, ≈30minutes after DBcAMP infusion). Furthermore, the chosen dose to highlight the evaluation of cAMP effect on insulin sensitivity was the 0.01mg/kg dose.

**Table 1:** Effect of different ipv doses of DBcAMP on arterial pressure and glycemia.

DBcAMP dose(mg/kg)	Arterial pressure before DBcAMP infusion (mmHg)	Arterial pressure after DBcAMP infusion (mmHg)	Glycemia before DBcAMP infusion (mg/dl)	Glycemia after DBcAMP infusion (mg/dl)
0.01	89.5±8.0	90.8±8.0	119.3±5.8	122.03±7.2
0.1	113.0±4.0	114.7±2.2	116.3±10.5*	126.7±14.2*
1	93.0±4.0	98.0±13.0	122.6±6.1*	187.8±25.3*

Results are means±SEM, n = 10,

*p<0.05.

**Fig 1:**
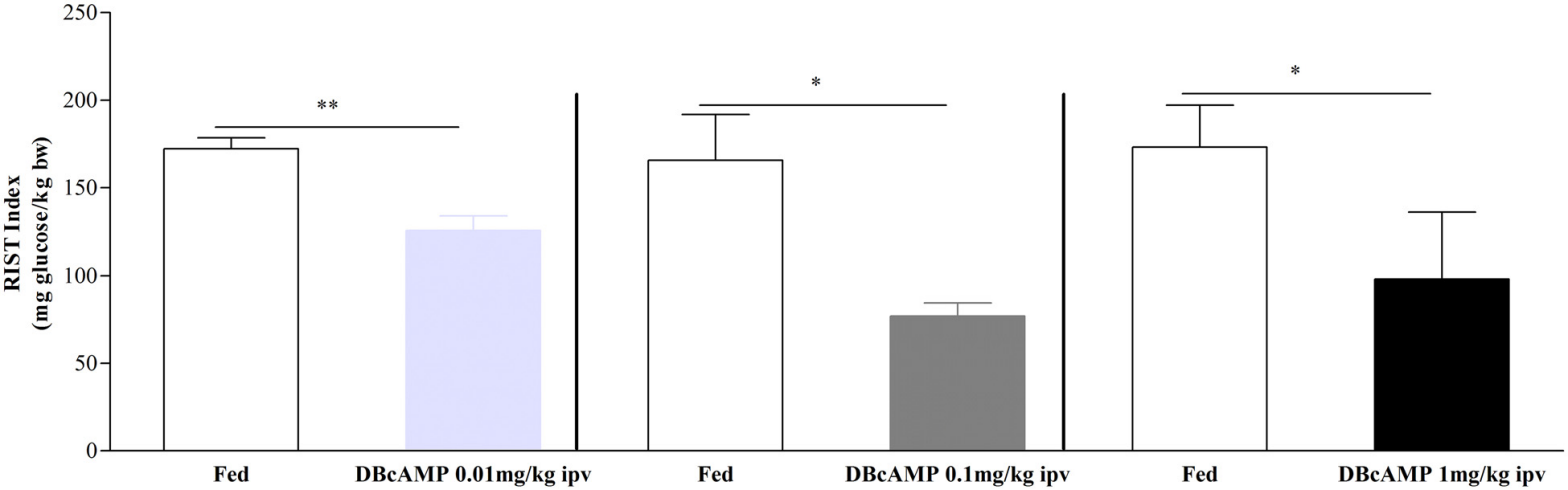
Effect of DBcAMP on insulin sensitivity. Insulin sensitivity decreases after DBcAMP 0.01, 0.1 and 1mg/kg ipv infusion. Results are means±SEM. Paired t-test. ** = p<0.01, * = p<0.05.

We found that increasing doses of ipv DBcAMP (n = 8) lead to decrease peripheral insulin sensitivity (DBcAMP 0.01mg/kg: from 172.3±6.3mg glucose/kg bw to 125.7±8.3mg glucose/kg bw, p<0.01; DBcAMP 0.1mg/kg: from 165.7±26.2 mg glucose/kg bw to 77.0±7.5mg glucose/kg bw, p<0.05; DBcAMP 1mg/kg: from 173.2±24.0mg glucose/kg bw to 98.1±38.0mg glucose/kg bw, p<0.05) (Fig 1). This decrease of peripheral insulin sensitivity corresponds to an inhibition of 27.2±2.1%, 51.6±12.2%, 47.2±14.1% for the DBcAMP 0.01, 0.1 and 1mg/kg doses, respectively. These results indicate that intraportal infusion of DBcAMP induced a state of insulin resistance suggesting that cAMP play a role in regulating whole-body glucose homeostasis.

### Glucagon action in the liver reduces postprandial insulin sensitivity

**Fig 2:**
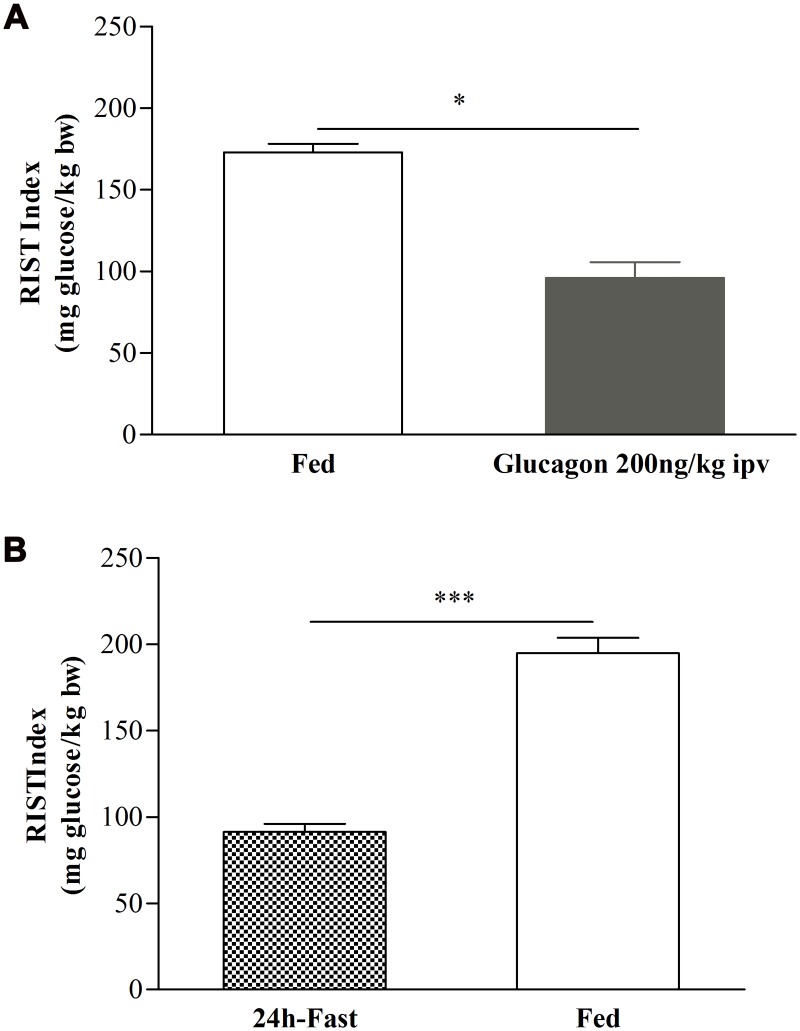
Effect of ipv glucagon 200ng/kg infusion and 24h-fast and fed on insulin sensitivity. Insulin sensitivity decreases after glucagon 200ng/kg ipv infusion(A) and insulin sensitivity increases in the fed state (B). Results are means±SEM, n = 4. Paired t-test. *** = p<0.001, * = p<0.05.

Since glucagon increases hepatic cAMP and affects glucose and insulin metabolism, we sought to evaluate the role of glucagon in postprandial insulin sensitivity. A dose-response infusion experiment was performed to evaluate the effect of different doses of glucagon (0.5, 1, 2.5, 5, 10, 200ng/kg, 2 and 20±g/kg, ipv, n = 14) on arterial pressure and arterial glycemia. The values of mean arterial pressure were not significantly changed by any of the glucagon doses infused (data not shown). Basal fed glycemia levels had a negligible increase in glycemia after glucagon 0.5, 1, 2.5, 5, 10 and 200ng/kg ipv infusion (S6A Fig), suggesting that in this dose range glucagon did not reach a threshold to increase glycemic levels. On the other hand, the higher doses of glucagon tested, 2 and 20μg/kg, induced a significant increase in glucose levels after infusion (S6B Fig). To measure the effect of glucagon infusion on postprandial insulin sensitivity, we choose the ipv glucagon dose of 200ng/kg. This dose represents the highest tested dose that did not have a sensible increase in basal glycemic levels at the time of performing the RIST (S1 Fig). As shown in Fig 2A, the RIST index decreased from 173.0±5.3mg glucose/kg bw to 96.1±9.7mg glucose/kg bw (n = 4, p<0.05), corresponding to a 42.6±6.5% inhibition of insulin sensitivity. This indicates that, intraportal glucagon infusion in the fed state was able to decrease insulin sensitivity to similar levels obtained in the fasted state (fasted to fed state: 91.3±4.8 and 194.9±8.8mg glucose/kg bw, respectively, n = 6, p<0.001, Fig 2B). Thus, we found that both DBcAMP and glucagon liver infusions were able to decrease postprandial insulin sensitivity. It is well-known that glucagon levels leads to increased hepatic cAMP levels [22] and our data suggests that glucagon effect in decreasing insulin sensitivity may operates through increasing cAMP hepatic levels and consequently decreased hepatic GSH levels [6]. The relevance of hepatic GSH levels in insulin sensitivity is highlighted by other studies where the use of L-buthionine-[*S*, *R*]-sulfoximine (BSO), which decreases hepatic GSH content, leads to an impairment of postprandial insulin sensitivity [1, 23].

### Nitric Oxide bioavailability and glucagon effects in insulin sensitivity

**Fig 3:**
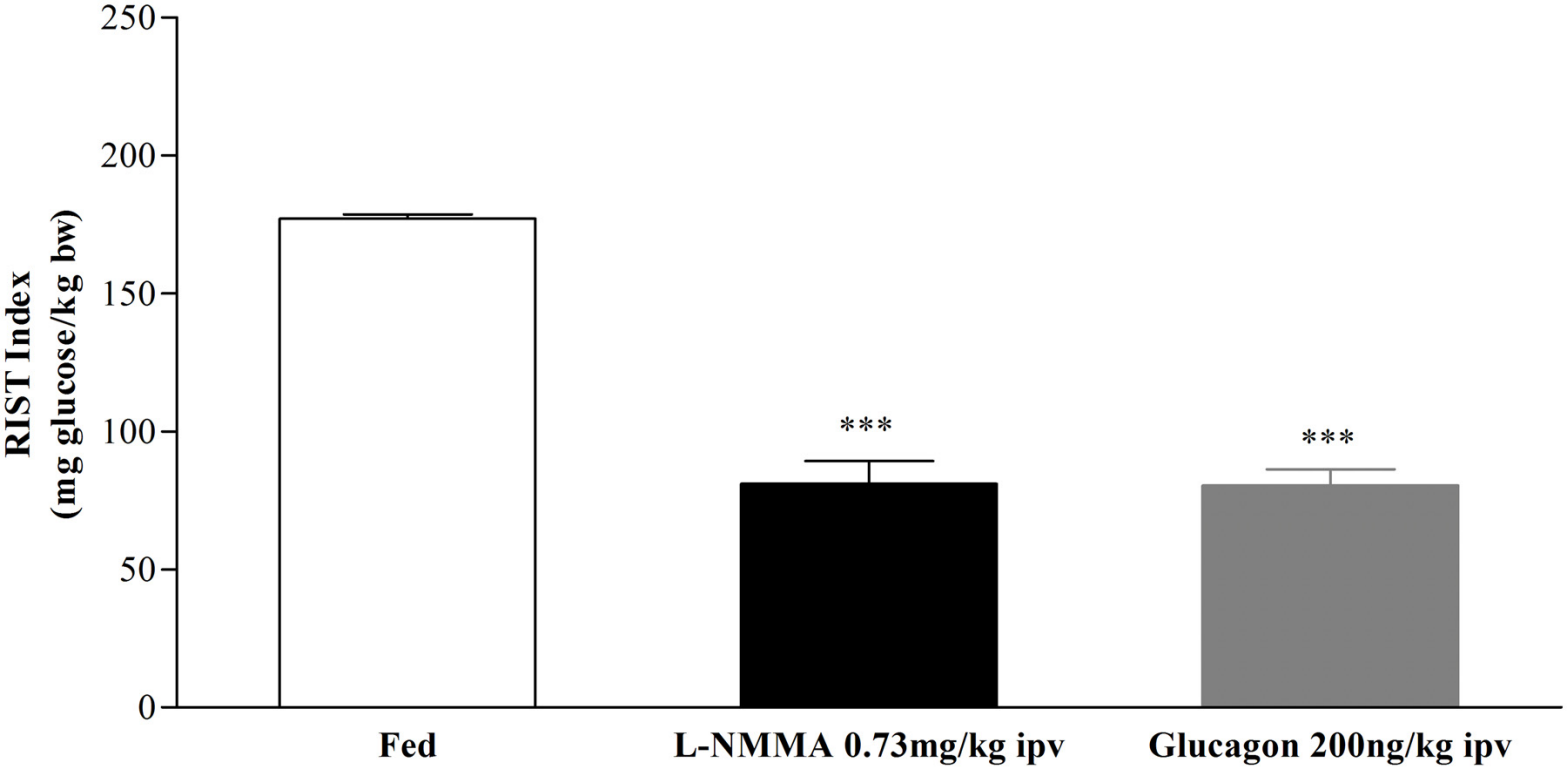
Effect of L-NMMA and glucagon on insulin sensitivity. The insulin sensitivity decreased after ipv L-NMMA 0.73mg/kg infusion and did not change after ipv glucagon 200ng/kg infusion. Results are means±SEM, n = 5. Repeated measures ANOVA, followed by the Tukey-Kramer multiple-comparison test. *** = p<0.001 Control *vs* L-NMMA 0.73mg/kg and Control *vs* Glucagon 200ng/kg.

Insulin sensitivity in the fed state is dependent on GSH and NO and we tested whether glucagon is affected by hepatic NO levels in decreasing insulin sensitivity. Therefore we evaluated if after inhibition by L-NMMA (a nitric oxide synthase specific inhibitor), glucagon was able to further affect postprandial insulin sensitivity. In this series of experiments, the control fed RIST index was 177.1±1.6mg glucose/kg bw and after ipv L-NMMA infusion the RIST index decreased to 81.0±8.3mg glucose/kg bw (n = 5, p<0.001), corresponding to a full strong inhibition of postprandial insulin sensitivity. Glucagon infusion did not further alter the RIST index (80.4±5.9mg glucose/kg bw, n = 5, p<0.001, Fig 3). Indeed, the percentages of inhibition of insulin sensitivity after administration of L-NMMA and glucagon were 54.2±4.9 and 54.6±3.5%, respectively. These results suggest that when postprandial insulin sensitivity was already impaired by low NO levels, glucagon does not promote an increase in the observed insulin resistance.

**Fig 4:**
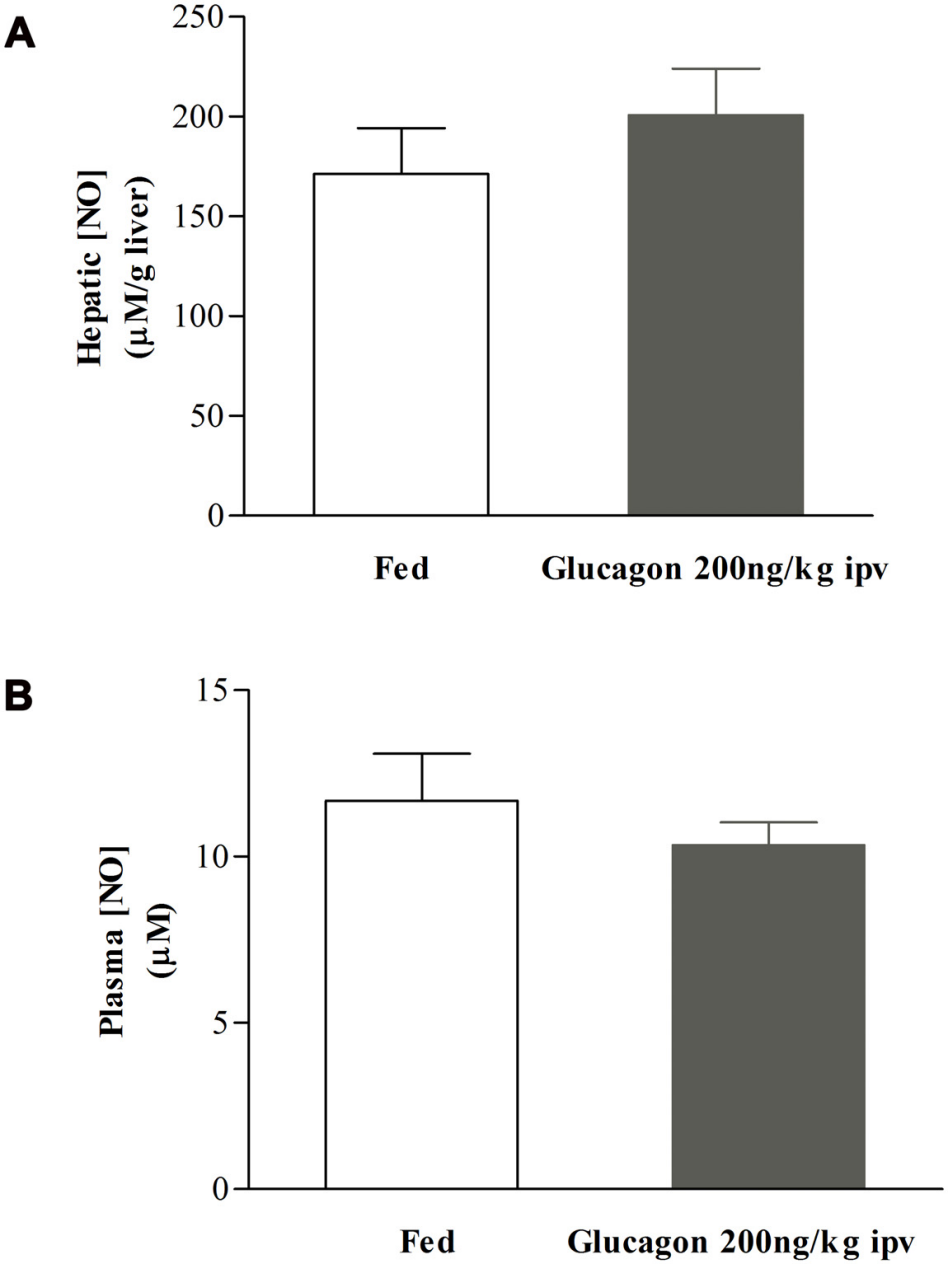
Effect of glucagon 200ng/kg on hepatic and plasma NO content. The hepatic NO content did not change after glucagon ipv 200ng/kg infusion in the liver (A) nor in the circulation (B). Results are means±SEM, n = 7. Unpaired t-test.

On the other hand, the hepatic and plasma levels of NO were not altered after acute ipv glucagon 200ng/kg infusion (hepatic levels: from 171.3±23.1 to 200.6±23.5μM/g liver, n = 7; plasma levels: from 11.7±1.4 to 10.3±0.7μM, n = 7, Fig 4A and 4B). These results suggested that acute ipv glucagon infusion did not affect hepatic NO bioavailability in regulation of postprandial insulin sensitivity. Together these data raise the possibility that glucagon-induced decrease of postprandial insulin sensitivity was not dependent on hepatic NO levels.

### Hepatic GSH is an intermediate of decreased insulin sensitivity mediated by glucagon

**Fig 5:**
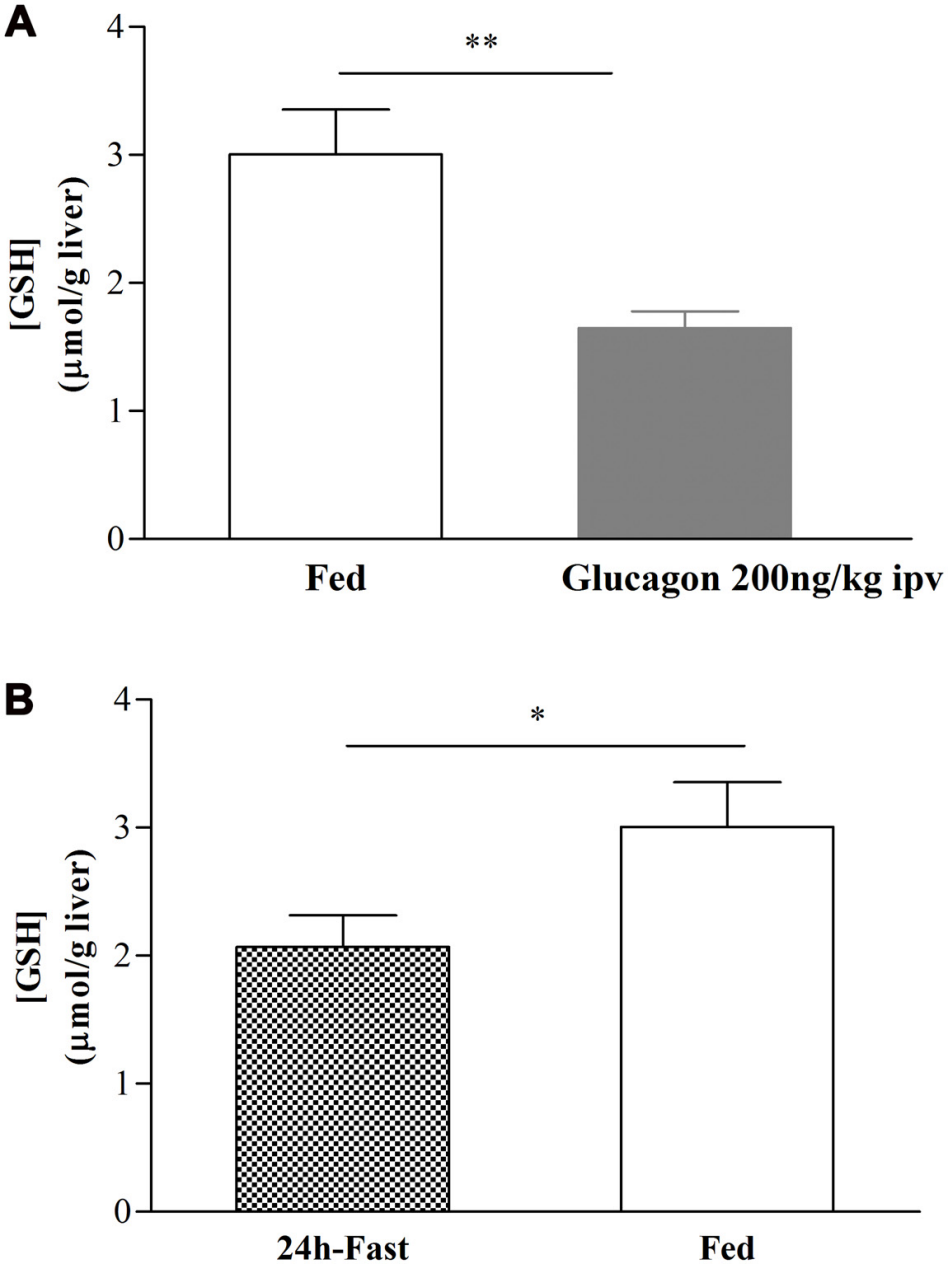
Effect of glucagon 200ng/kg on hepatic GSH content. The hepatic GSH content decreases after glucagon ipv 200ng/kg infusion (A) to levels obtained in the control fast situation (B). Results are means±SEM, n = 7. Unpaired t-test. ** = p<0.01, * = p<0.05.

**Fig 6:**
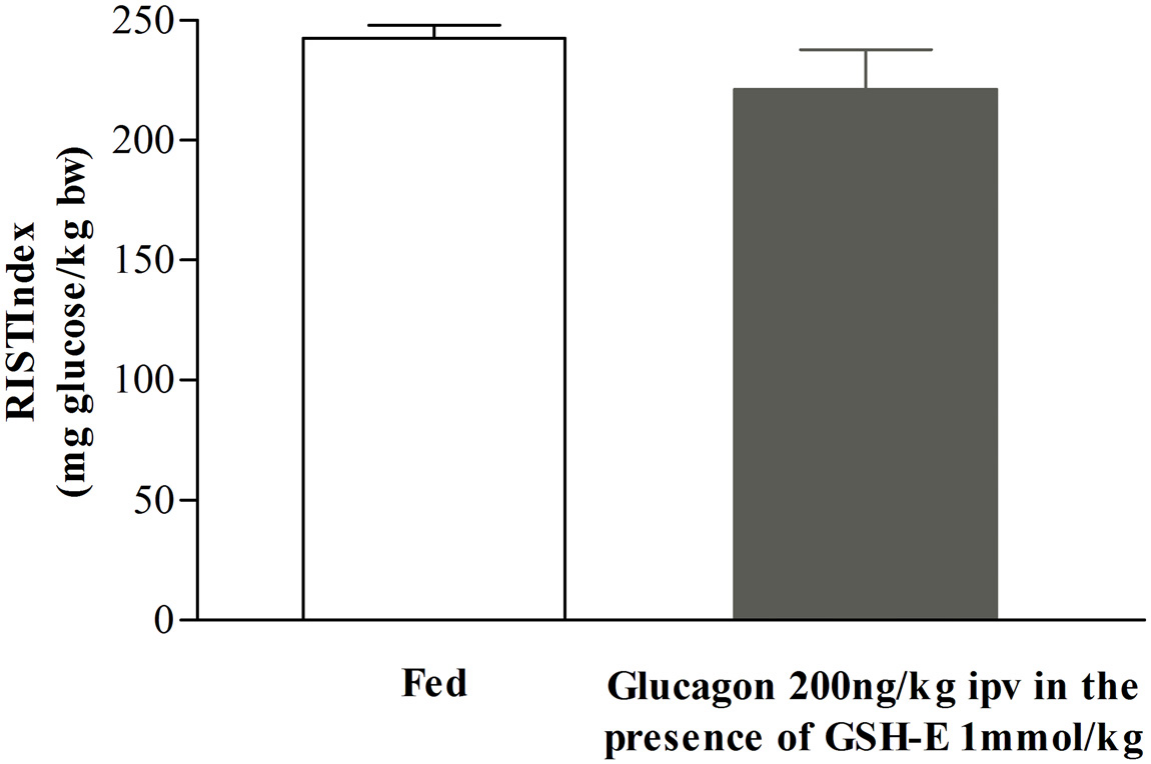
Effect of GSH-E and glucagon on insulin sensitivity. The insulin sensitivity did not change when ipv glucagon 200ng/kg infusion was given after GSH-E 1mmol/kg ipv infusion. Results are means±SEM, n = 3.

**Fig 7:**
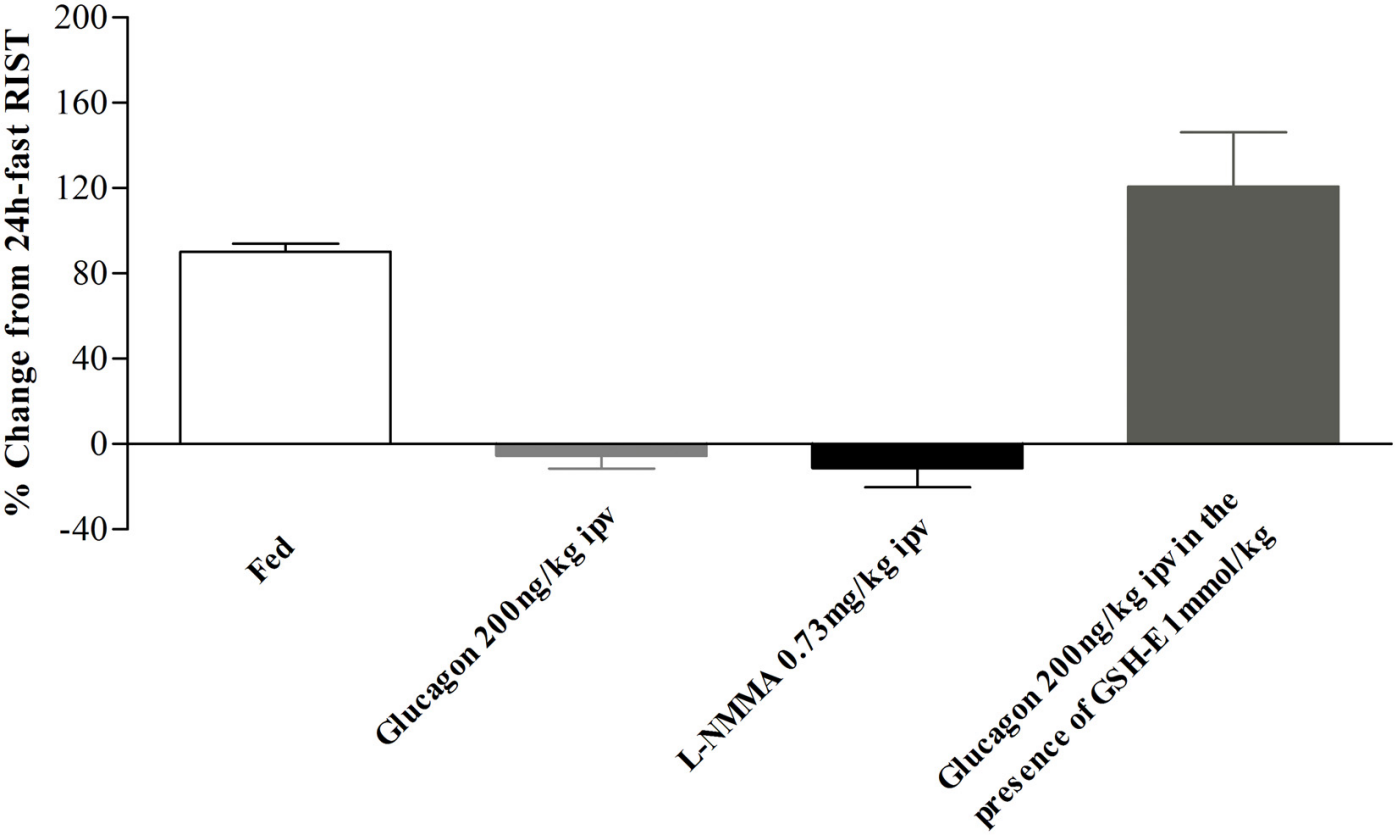
The insulin resistance induced either by ipv glucagon and L-NMMA infusion was reversed by glucagon in the presence of GSH-E. Results are means±SEM.

It was described by Lu et al. [6] a relationship between hepatic GSH levels and glucagon, through the increase of hepatic cAMP levels. We observed that after ipv glucagon 200ng/kg infusion, hepatic GSH content decreased in comparison to the fed levels (from 3.0±0.4 to 1.7±0.1μmol/g liver, n = 7, Fig 5A) and is similar to control fasted animals (2.1±0.3μmol/g liver, n = 4, Fig 5B). To test whether the effect of glucagon on GSH hepatic content was required to decrease insulin sensitivity we made use of GSH-E (a GSH donor/agonist) that is effectively transported into hepatocytes converted into GSH [24] and able to enhance insulin sensitivity when optimal hepatic NO levels were present [5]. We found that the post-glucagon 200ng/kg ipv RIST index after GSH-E ipv infusion (221.4μ16.5mg glucose/kg, n = 3) was not different from the control fed RIST index (242.7α5.4mg glucose/kg, n = 3) (Fig 6). This indicates that GSH-E was able to restore insulin sensitivity after its inhibition by glucagon infusion. In Fig 7 it was represented the percentage of change from the 24h-fast RIST after all the drugs used. Glucagon 200ng/kg and L-NMMA 0.73mg/kg showed a complete inhibition of insulin sensitivity. Glucagon infusion followed by GSH-E administration leads to a complete restoration of insulin sensitivity, previously inhibited by ipv glucagon 200ng/kg infusion.

In summary, we showed that glucagon 200ng/kg showed a complete inhibition of insulin sensitivity while GSH-E administration after ipv glucagon infusion leads to a complete restoration of insulin sensitivity. This work identifies a role for glucagon in reducing postprandial insulin sensitivity and unravels that it operates through decreasing GSH hepatic levels.

## Discussion

The increase in postprandial insulin sensitivity is dependent on the activation of the hepatic parasympathetic nerves, leading to an increase in hepatic NO and GSH levels [4, 5]. It was also observed that this increase in postprandial insulin sensitivity was not due to a change in peripheral insulin levels [25]. These observations led to a new hypothesis that involves the liver as a key regulator in the modulation of peripheral insulin sensitivity, mainly at skeletal muscle, kidney and heart through the release of an hepatic factor [1–3]. On this basis, this study aimed to understand the highly regulated pathway involved in the GSH synthesis, controlled by 3’,5’-cyclic adenosine 5’-monophosphate (cAMP) and/or glucagon action, and its repercussions on decreased postprandial insulin sensitivity. It has been reported that glucagon increases intracellular cAMP concentrations which in turn, induces a decrease in hepatic GSH, due to a decrease in activity of -glutamylcysteine synthase [6]. Together, our findings support the hypothesis that hyperglucagonemia acts via adenylate cyclase to decrease hepatic GSH levels, leading to an impairment in postprandial insulin sensitivity and to overall insulin resistance, a mechanism that would account for the development of postprandial hyperglycemia and type 2 diabetes.

### Hepatic cAMP/GSH and postprandial insulin sensitivity

Our results showed that glucagon infusion causes a state of decreased postprandial insulin sensitivity possibly mimicking the fasting state with respect to hepatic cAMP and GSH levels (Fig 5). Moreover, induction of postprandial insulin resistance by glucagon was overcome by restoring the levels of GSH, indicating that depletion of hepatic GSH is a critical mechanism of glucagon action in inducing insulin resistance (Fig 6). Although this action may require physiological levels of NO, glucagon does not have a measurable effect role on NO concentration, because acute ipv infusion of glucagon did not impact on hepatic or plasma levels of NO (Fig 4).

The effect of glucose on insulin secretion can be amplified by signaling pathways involving inositol trisphosphate and diacylglycerol, derived from activation of phospholipase C [26, 27], and by cAMP, following activation of adenylate cyclase [26]. In the liver, cAMP plays a different role and did not affect insulin levels (S3, S4 and S5 Figs). Here we showed that both ipv DBcAMP and glucagon infusions decreased insulin sensitivity (Figs 1 and 2). Under the experimental conditions insulin sensitivity was not influenced by insulin, since plasma insulin levels were not different before and after RIST (S3, S4 and S5 Figs). Therefore, DBcAMP did not impact on insulin levels and it is plausible that by increasing hepatic cAMP levels led to a decrease of GSH and consequently to a state of postprandial insulin resistance. Intracellular cAMP levels depend upon the balance between its formation through the activity of adenylate cyclase and its destruction by cAMP-degrading enzymes known as phosphodiesterases (PDEs). The activity of two of these cAMP-PDEs (PDE2A and PDE3B) in the liver is dependent on cGMP activation [28]. We have shown that hepatic NO/cGMP is essential for the regulation of the postprandial insulin sensitivity [4], which strongly suggests that cGMP-dependent cAMP-PDEs could represent fine regulators of the postprandial insulin sensitivity, by controlling GSH levels through a cAMP/cGMP crosstalk [29, 30]. The proposed mechanism is independent of hepatic NO regulation by glucagon as this hormone did not change NO levels after acute ipv infusion. Moreover, blocking postprandial insulin sensitivity with L-NMMA with a subsequent ipv infusion of glucagon did not aggravate the insulin resistance in fed animals (Fig 3). These results showed that glucagon caused a state of postprandial insulin resistance by decreasing GSH levels but did not operate through impacting on hepatic NO levels.

It has been suggested that decreased GSH levels operates to increase insulin sensitivity in the muscle in the fasting conditions [31, 32]. In contrast, we showed that in the fed state, GSH plays a pivotal role in the liver to induce increased peripheral insulin sensitivity [5, 23]. The fact that administration of GSH abrogates the glucagon effect on peripheral insulin sensitivity strongly suggests that GSH is an actor in mediating the observed glucagon effect.

The finely tuned balance of the two major pancreatic hormones, insulin and glucagon, is impaired in type 2 diabetic subjects. This is in agreement with an imbalance of the insulin:glucagon molar ratio, since this ratio mainly affects hepatic glucose production [33]. Because of the reduction of insulin:glucagon molar ratio, basal endogenous glucose concentration will be higher, causing fasting hyperglycemia and contributing to excessive postprandial glucose rise [34]. We can not exclude that glucagon acts in endogenous glucose production but under our experimental conditions this effect should be minimal or negligible. We do not anticipate that glucose appearance rate was influenced by glucagon infusion as the hormone was given at very low doses directly into the liver and arterial glycemia did not show sensible increasing before, during and after glucagon infusion (S1 Fig). Likewise we do not expect our glucagon administration protocol would have a sizable impact on endogenous glucose production mediating changes on postprandial insulin sensitivity since the liver only accounts for 7% of whole-body glucose clearance while the skeletal muscle contributes with 69% [3]. We further propose that high levels of glucagon in the postprandial state, could compromise insulin sensitivity, due to a decrease of hepatic GSH levels. This impairment on postprandial insulin sensitivity would aggravate the insulin resistance state, leading to increased glucose excursions.

### Hyperglucagonemia and Diabetes

Type 2 diabetes patients feature a bihormonal disorder where either absolute insulin insufficiency or relative lack of insulin is present alongside fasting and postprandial hyperglucagonemia [35]. It is important to note that glucagon levels are abnormally high in the specific context of hyperglycemia and hyperinsulinemia, whereas in untreated type 2 diabetes the level is sometimes not elevated in absolute terms [17]. Interestingly, it has recently been reported that the well-known disturbed pulsatility of insulin secretion in type 2 diabetes [36] is concomitant with disturbed glucagon pulsatility (higher pulse mass in patients with type 2 diabetes), possibly contributing to the hyperglucagonemia in these patients [37].

In the past decades increasing evidence, including various interventions targeting glucagon secretion, glucagon’s receptor and glucagon clearance, has emerged to unequivocally support the role of fasting and postprandial hyperglucagonemia, as major contributing factor for the elevated levels of blood glucose, a hallmark of diabetes [17, 38–41]. Whereas, the mechanisms underlying hyperglucagonemia in fasting and in postprandial state are not entirely identical their pathophysiological effects could be therapeutically targeted by repression of glucagon secretion/action throughout the day. In this respect, our data suggests that sustained high levels of glucagon, usually seen in type 2 diabetes, may lead to a decline in hepatic GSH levels that decrease postprandial insulin sensitivity, culminating in a state of insulin resistance.

**Fig 8:**
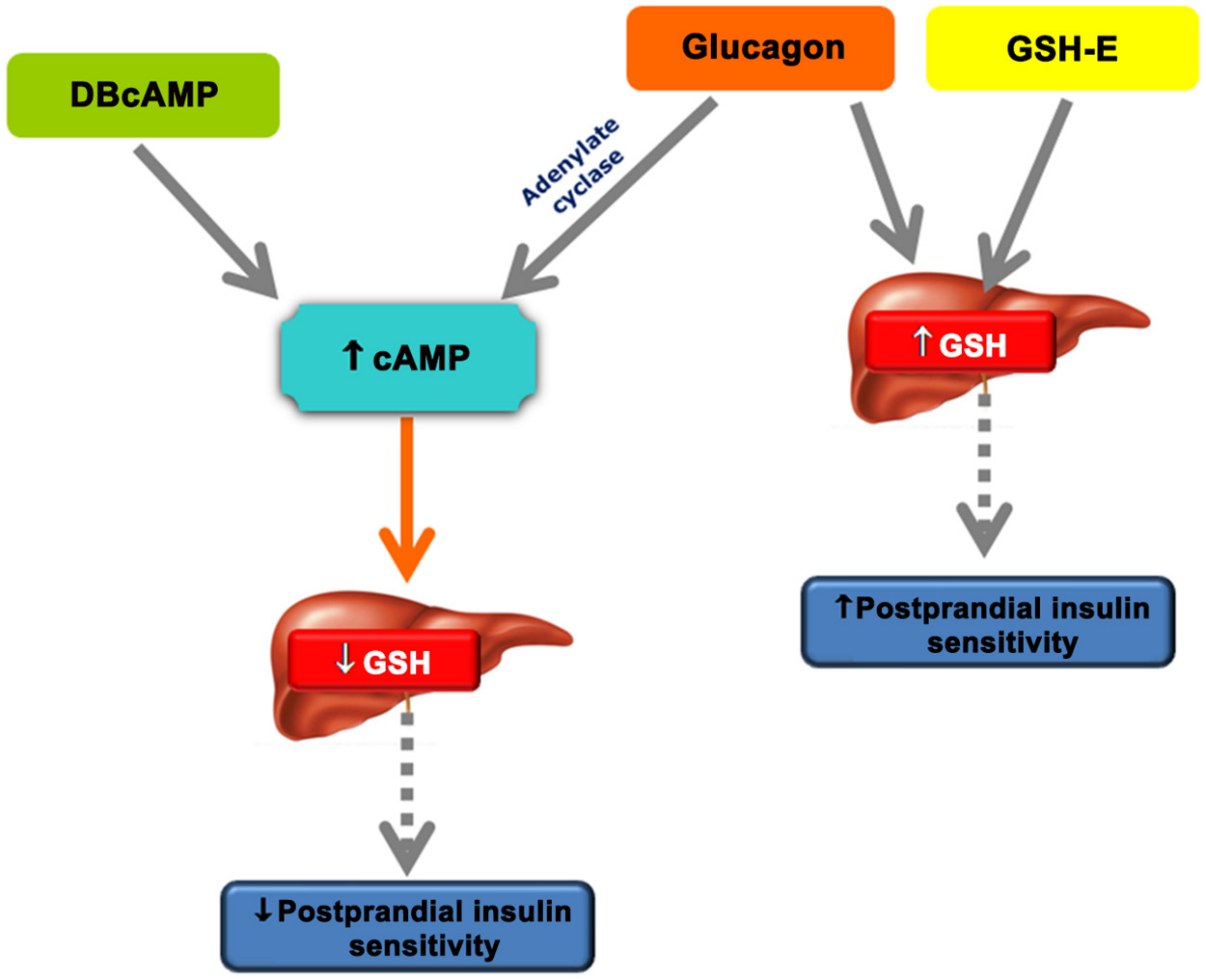
DBcAMP and glucagon produces a decrease of peripheral insulin sensitivity. The cAMP analog and glucagon promote an increase in hepatic cAMP levels that are related to the decrease of hepatic GSH synthesis, leading to an impairment of peripheral postprandial insulin sensitivity. On the other hand, in the presence of increased GSH levels the glucagon is abrogated and peripheral insulin sensitivity is restored. DBcAMP: N6,2‟-O-dibutyryladenosine 3‟,5‟-cyclicmonophosphate; cAMP: 3‟,5‟-cyclic adenosine 5‟-monophosphate; GSH:gluthatione.

This work supports for the first time the notion that glucagon controls postprandial insulin sensitivity through its inhibitory action on hepatic GSH formation. Furthermore, we suggest this effect is mediated through increasing cAMP levels. Insulin resistance is an early feature of diabetes progression and our results call attention for further studies addressing the axis glucagon-cAMP-GSH as new therapeutic target for the treatment of insulin resistant states, a hallmark of type 2 diabetes and obesity (Fig 8).

## Supporting Information

S1 Fig Glycemic profiles before RIST after glucagon infusion. Glycemic profiles at specific time points determined after saline and glucagon 200ng/kg ipv infusion (n = 4). Results are means±SEM.
(TIF)

S2 Fig Glycemic profiles before RIST after DBcAMP infusion. Glycemic profiles at specific time points determined after saline and DBcAMP 0.01, 0.1 and 1mg/kg ipv infusion (n = 10). Results are means±SEM.
(TIF)

S3 Fig Effect of DBcAMP 0.01mg/kg on plasma insulin levels. Insulin levels were not altered by ipv DBcAMP infusion (0.01mg/kg). Results are means±SEM. One-way ANOVA, followed by the Tukey-Kramer multiple-comparison test.
(TIF)

S4 Fig Effect of DBcAMP 0.1mg/kg on plasma insulin levels. Insulin levels were not altered by ipv DBcAMP infusion (0.1mg/kg). Results are means±SEM. One-way ANOVA, followed by the Tukey-Kramer multiple-comparison test.
(TIF)

S5 Fig Effect of DBcAMP 1mg/kg on plasma insulin levels. Insulin levels were not altered by ipv DBcAMP infusion (1mg/kg). Results are means±SEM. One-way ANOVA, followed by the Tukey-Kramer multiple-comparison test.
(TIF)

S6 Fig Glycemic profile at specific time points determined after glucagon 0.5, 1, 2.5, 5, 10, 200ng/kg, 2 and 20μg/kg ipv infusion. The lowest ipv glucagon doses had a minimal or negligible effect on glycemia **(A)** on the other hand, doses of 2 and 20μg/kg promoted a significantly increase in glycemia **(B)**. Results are means±SEM, n = 14.
(TIF)
