# Supplementary figures and images for: Acute Glucagon Induces Postprandial Peripheral Insulin Resistance
Source: PLoS One. 2015 May 11;10(5):e0127221. doi: 10.1371/journal.pone.0127221 (PMC4427479; doi:10.1371/journal.pone.0127221)

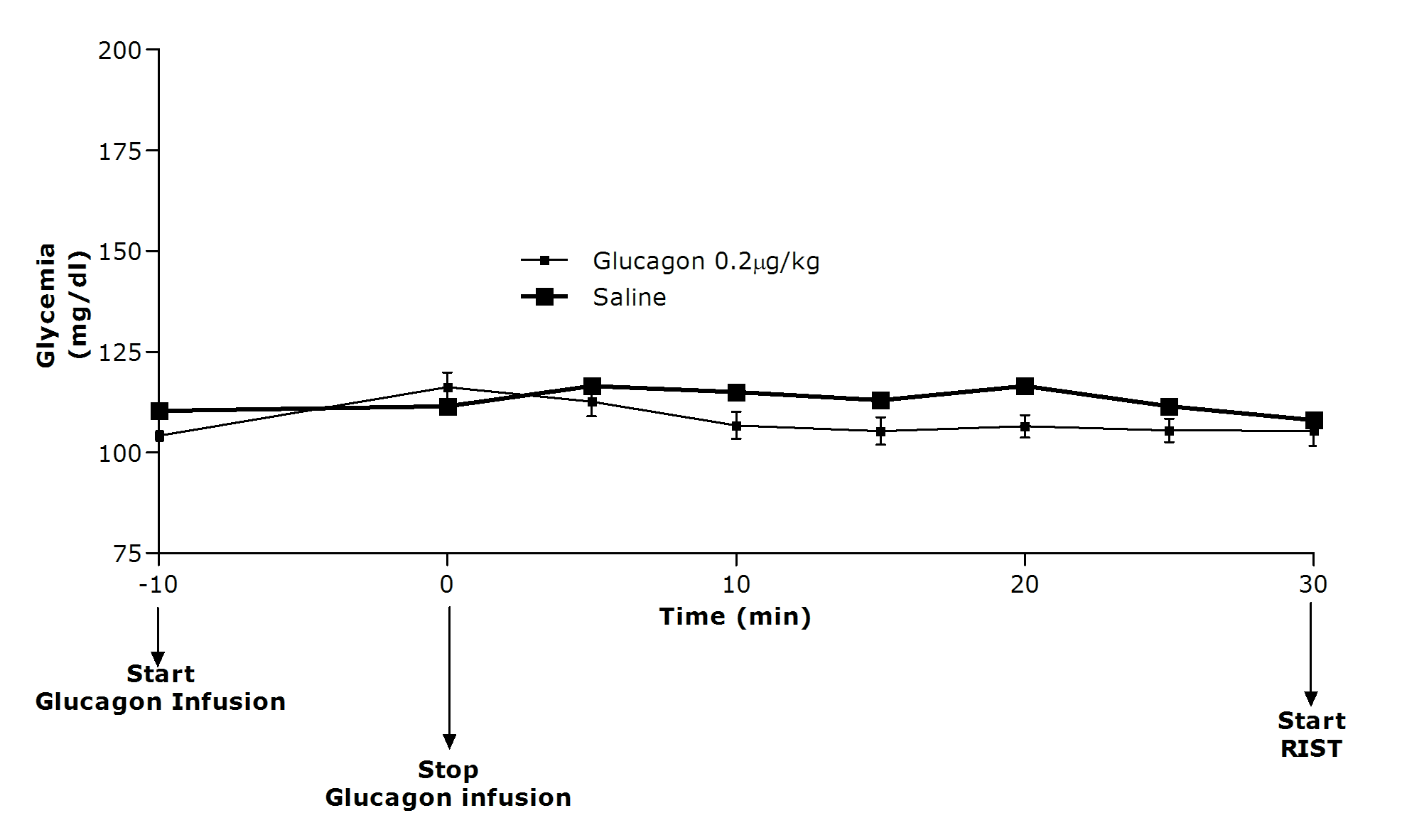

Supplement: S1 Fig — Glycemic profiles at specific time points determined after saline and glucagon 200ng/kg ipv infusion (n = 4). Results are means±SEM. (TIF) [file pone.0127221.s001.tif]

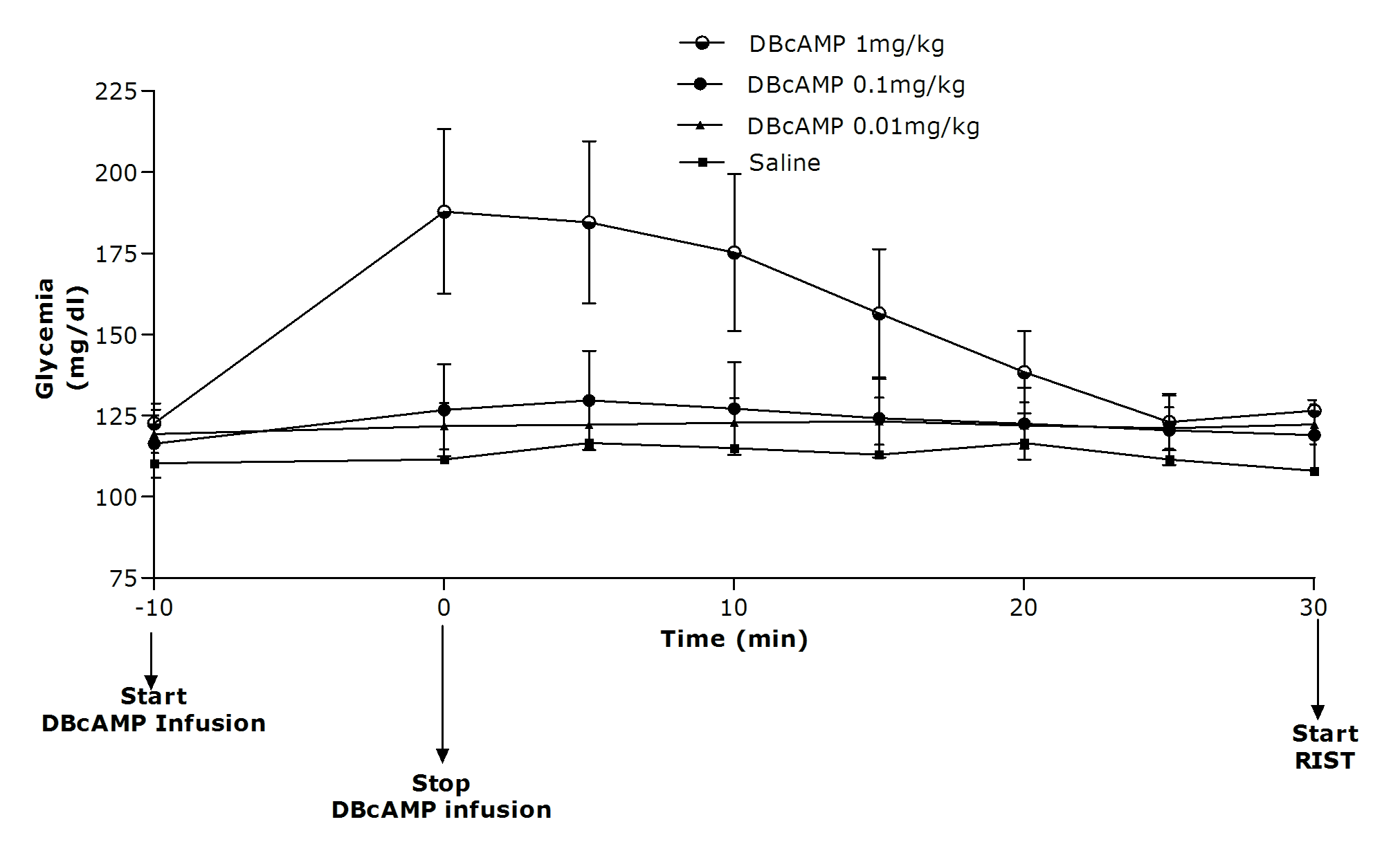

Supplement: S2 Fig — Glycemic profiles at specific time points determined after saline and DBcAMP 0.01, 0.1 and 1mg/kg ipv infusion (n = 10). Results are means±SEM. (TIF) [file pone.0127221.s002.tif]

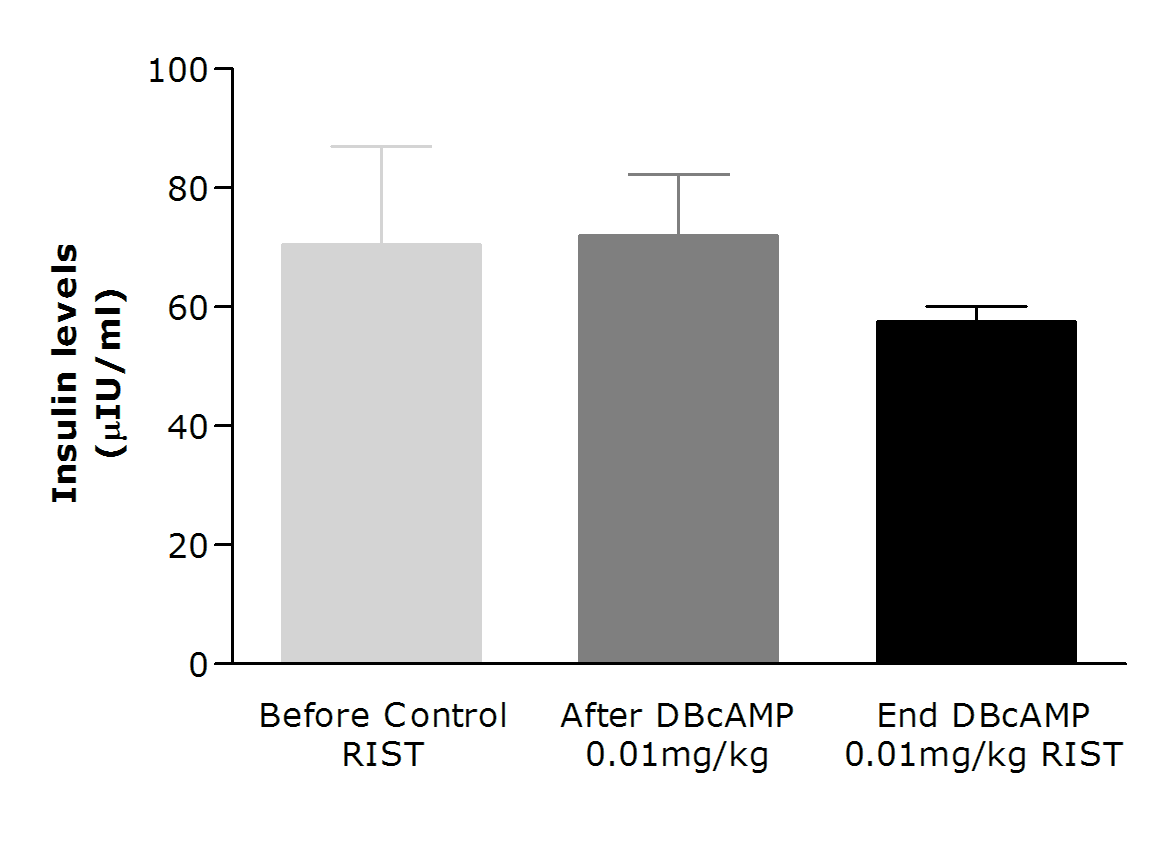

Supplement: S3 Fig — Insulin levels were not altered by ipv DBcAMP infusion (0.01mg/kg). Results are means±SEM. One-way ANOVA, followed by the Tukey-Kramer multiple-comparison test. (TIF) [file pone.0127221.s003.tif]

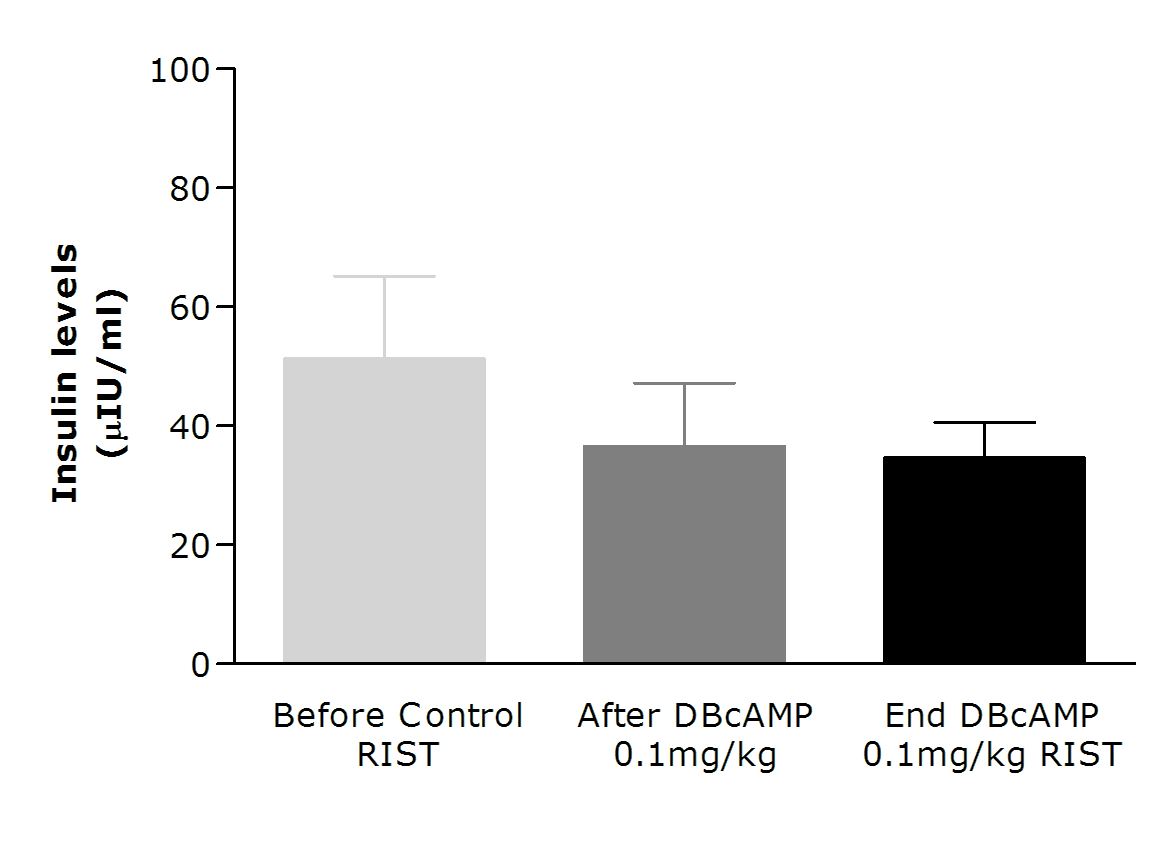

Supplement: S4 Fig — Insulin levels were not altered by ipv DBcAMP infusion (0.1mg/kg). Results are means±SEM. One-way ANOVA, followed by the Tukey-Kramer multiple-comparison test. (TIF) [file pone.0127221.s004.tif]

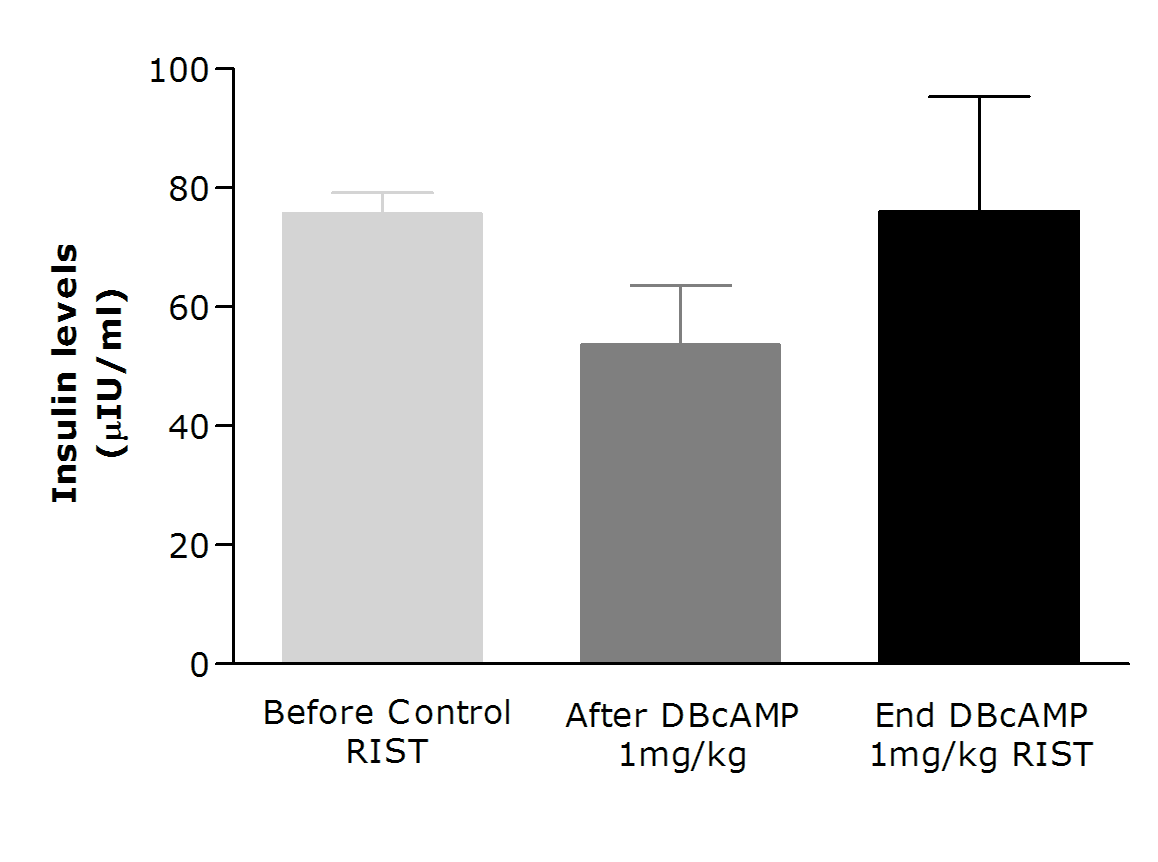

Supplement: S5 Fig — Insulin levels were not altered by ipv DBcAMP infusion (1mg/kg). Results are means±SEM. One-way ANOVA, followed by the Tukey-Kramer multiple-comparison test. (TIF) [file pone.0127221.s005.tif]

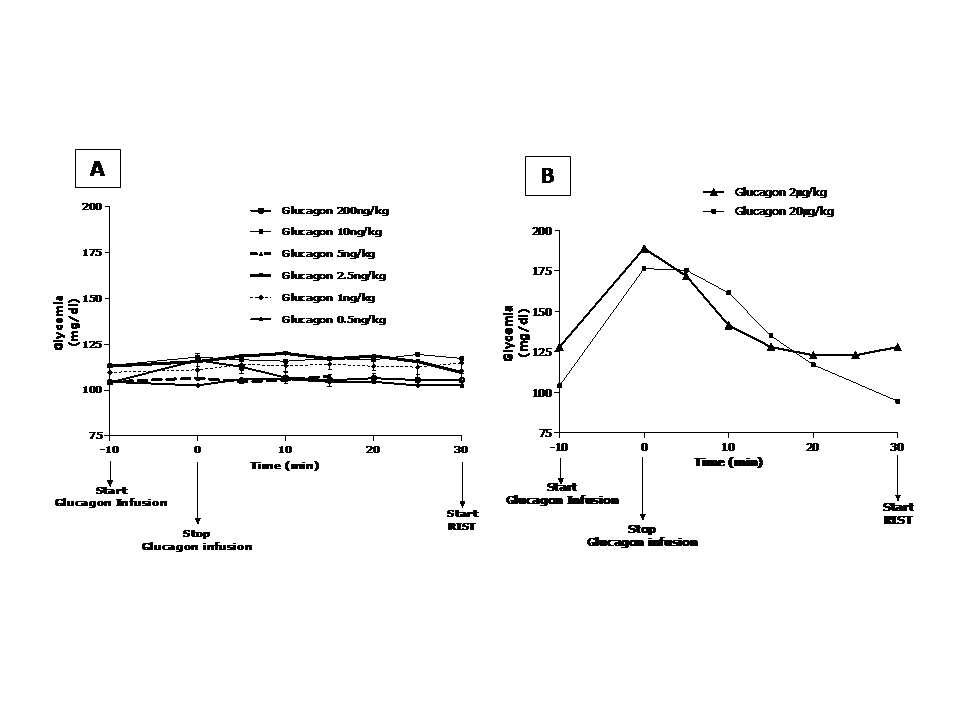

Supplement: S6 Fig — The lowest ipv glucagon doses had a minimal or negligible effect on glycemia (A) on the other hand, doses of 2 and 20μg/kg promoted a significantly increase in glycemia (B). Results are means±SEM, n = 14. (TIF) [file pone.0127221.s006.tif]
